# Rehabilitation and Parkinson's Disease 2013

**DOI:** 10.1155/2013/506375

**Published:** 2013-08-28

**Authors:** Gammon M. Earhart, Leland E. Dibble, Terry Ellis, Alice Nieuwboer

**Affiliations:** ^1^Program in Physical Therapy, Department of Anatomy and Neurobiology and Department of Neurology, Washington University in St. Louis School of Medicine, St. Louis, MO 63108, USA; ^2^Department of Physical Therapy, University of Utah, Salt Lake City, UT 84108, USA; ^3^Department of Physical Therapy and Athletic Training, Boston University, Boston, MA 02215, USA; ^4^Department of Rehabilitation Sciences, Katholieke Universiteit Leuven, 3001 Leuven, Belgium

Parkinson's disease (PD) is the second most common neurodegenerative disorder, and increasing age is a major risk factor for development of PD. As such, PD is expected to become increasingly prevalent in coming years as the aged population grows in number. PD is typically treated with pharmacological and sometimes surgical approaches, but these treatments do not adequately address many aspects of the disease. As such, rehabilitation may play a key role in the management of PD. The nine articles in this special issue illustrate the broad spectrum of important rehabilitation issues for people with PD. M. H. Nilsson et al. report relationships between health and housing in very old individuals with PD, examining the impact of environmental barriers and accessibility problems on daily life. A. Letanneux et al. focus on the psychosocial impact of speech impairment in PD, presenting a French version of the Dysarthria Impact Profile. K. B. Foreman et al. also address speech issues, examining the effects of concurrent performance of a speech task and a postural control task in individuals with PD. Postural control is also addressed by G. Vervoort et al., who present evidence of differences in specific aspects of postural control in people with PD who experience freezing of gait compared to those with PD who have no history of freezing of gait. S. T. Nemanich et al. also focus on gait in PD, examining utility of walking speeds for identifying fallers and determining predictors of preferred and fast pace walking speeds. A. Williams et al. examine the relationships between gait and upper extremity movements in PD, studying the effects of amplitude and cadence manipulations. B. K. Randhawa et al. also examine upper extremity performance as assessed by handwriting, demonstrating the acute changes in handwriting following a single intervention session using transcranial magnetic stimulation. L. A. King et al. examine two different exercise interventions, focusing on which outcome measures were most effective for measuring change following agility boot camp or treadmill training. Finally, G. Frazzitta et al. report the effects of a four-week multidisciplinary inpatient rehabilitation program on gait and balance function after completion of the intervention and one year later. The broad scope of work in this special issue is reflective of the far-reaching impact that rehabilitation may have on many aspects of PD, from the individual to the environmental level. Last but not least, the presented work will provide a multilevel understanding of PD motor problems which will feed into the clinical care and optimal rehabilitation for patients with this complex disease. 

*Gammon M. Earhart* *Gammon M. Earhart* *Leland E. Dibble* *Leland E. Dibble* *Terry Ellis* *Terry Ellis* *Alice Nieuwboer* *Alice Nieuwboer*

